# Supplementary material for: Biological and Cellular Properties of Advanced Platelet-Rich Fibrin (A-PRF) Compared to Other Platelet Concentrates: Systematic Review and Meta-Analysis
Source: Int J Mol Sci. 2023 Dec 29;25(1):482. doi: 10.3390/ijms25010482 (PMC10779223; doi:10.3390/ijms25010482)
Supplement: Supplementary file 1 [file ijms-25-00482-s001.zip › ijms-2752785-supplementary.pdf]

Table S1. Risk of bias the included manuscripts.

**Criterial**	Ghanaati et al, 2014	Kobayashi et al, 2016	Masuki et al, 2016	Fugioka-Kobayashi et al, 2016	Lei et al, 2020	Pitzurra et al, 2020	Esfahood et al, 2020
Clearly stated aims/objectives	**Adequately Specified (2)**	**Adequately Specified (2)**	**Adequately Specified (2)**	**Adequately Specified (2)**	**Adequately Specified (2)**	**Adequately Specified (2)**	**Adequately Specified (2)**
Detailed explanation of sample size calculation	**Not Applicable**	**Not Applicable**	**Not Applicable**	**Not Applicable**	**Not Applicable**	**Not Applicable**	**Not Applicable**
Detailed explanation of sampling technique	**Adequately Specified (2)**	**Adequately Specified (2)**	**Adequately Specified (2)**	**Adequately Specified (2)**	**Adequately Specified (2)**	**Adequately Specified (2)**	**Adequately Specified (2)**
Details of comparison group	**Adequately Specified (2)**	**Adequately Specified (2)**	**Adequately Specified (2)**	**Adequately Specified (2)**	**Adequately Specified (2)**	**Adequately Specified (2)**	**Adequately Specified (2)**
Detailed explanation of methodology	**Adequately Specified (2)**	**Adequately Specified (2)**	**Adequately Specified (2)**	**Adequately Specified (2)**	**Adequately Specified (2)**	**Adequately Specified (2)**	**Adequately Specified (2)**
Operator details	**Not Applicable**	**Not Applicable**	**Not Applicable**	**Not Applicable**	**Not Applicable**	**Not Applicable**	**Not Applicable**
Randomization	**Not Applicable**	**Not Applicable**	**Not Applicable**	**Not Applicable**	**Not Applicable**	**Not Applicable**	**Not Applicable**
Method of measurement of outcome	**Adequately Specified (2)**	**Adequately Specified (2)**	**Adequately Specified (2)**	**Adequately Specified (2)**	**Adequately Specified (2)**	**Adequately Specified (2)**	**Adequately Specified (2)**

Outcome assessor details	**Adequately Specified (2)**	**Adequately Specified (2)**	**Adequately Specified (2)**	**Adequately Specified (2)**	**Adequately Specified (2)**	**Adequately Specified (2)**	**Adequately Specified (2)**
Blinding	**Not Applicable**	**Not Applicable**	**Not Applicable**	**Not Applicable**	**Not Applicable**	**Not Applicable**	**Not Applicable**
Statistical analysis	**Adequately Specified (2)**	**Adequately Specified (2)**	**Adequately Specified (2)**	**Adequately Specified (2)**	**Adequately Specified (2)**	**Adequately Specified (2)**	**Adequately Specified (2)**
Presentation of results	**Adequately Specified (2)**	**Adequately Specified (2)**	**Adequately Specified (2)**	**Adequately Specified (2)**	**Adequately Specified (2)**	**Adequately Specified (2)**	**Adequately Specified (2)**
**Total Score**	62,5	62,5	62,5	62,5	62,5	62,5	62,5
**Conclusion**	Medium Risk of Bias	Medium Risk of Bias	Medium Risk of Bias	Medium Risk of Bias	Medium Risk of Bias	Medium Risk of Bias	Medium Risk of Bias

LEGEND: >70% - Low risk of bias; 50 to 70% - Medium risk of bias; <50% - High risk of bias. Adequately Specified (Score=2); Inadequately Specified (Score=1); Not Specified (Score=0); Not Applicable.

Table S2. Excluded studies and reasons for exclusion.

Excluded Studies	Reasons for Exclusion
**Fernandez-Medina et al, 2019**	Use of animal sample
**Castro et al, 2021**	Non-experimental studies
**Kartika et al, 2021**	Non-experimental studies
**Clark et al, 2018**	Non-experimental studies
**Ivanova et al, 2019**	Non-experimental studies
**Kalash et al, 2017**	Non-experimental studies
**Zahid et al, 2019**	Non-experimental studies
**Yuce et al, 2019**	Non-experimental studies

Table S3. Description of data on the use of A-PRF for regeneration tissue.

Author, year/ country	Cell culture	Intervention	Groups: C, control ǀ T, test	Protocol Preparation
Ghanaati et al., (2014)<br>Germany, United States, France	Fibrin-based clots fixed paraformaldehyde (4%)	Cassettes of fibrine clots was analyzed for histological evaluation, histomorphometry and immunohistochemistry	C, L-PRF; and<br>T, A-PRF	Blood was collected for four heathy volunteers.<br>A-PRF: 200 G for 14 minutes<br>L-PRF: 700 G for 12 minutes
Kobayashi et al, (2016)<br>Switzerland, Japan	A-PRF and PRP, PRF was isolate and fixed for analyse	In order to determine the amount of released growth factors samples were placed into a shaking incubator at 37 °C to allow for growth factor release into the culture media	C[1], PRP; and<br>T, A-PRF; and<br>C[2], PRF	Three samples of blood were collected of six volunteer donors<br>A-PRF: 200 G for 14 minutes<br>PRP: 112 G for 7 minutes, after plasma decanted 1000 G for 10 minutes<br>PRF: 700 G for 12 minutes

Masuki et al, (2016)<br><br>Japan	Human alveolar bone-derived periosteal cells	The numbers of blood cells were determined using automated hematology analyzer	C[1], PRP; and<br><br>T[1], A-PRF; and<br><br>C[2], CGF<br><br>C[3], PRGF	The blood collected for the seven healthy volunteers<br><br>A-PRF: 198 G for 8 minutes<br><br>PRP: 1150 G for 4 minutes in two times<br><br>PRGF: 580 G for 8 minutes<br><br>CGF: 645 G for 4 minutes
Fujioka – Kobayashi et al, (2016)<br><br>EUA	Viabilidade e migração de fibroblastos gengivais humanos	Comparação e quantificação de Proteína com Ensaio de Imunoabsorção Enzimática (ELISA)	C, L-PRF; and<br><br>T[1], A-PRF; and<br><br>T[2], A-PRF+	Sangue coletado de oito voluntários saudáveis<br><br>A-PRF: 200 G for 14 minutes<br><br>L-PRF: 700 G for 12 minutes<br><br>A-PRF+: 200 G for 8 minutes
Lei et al, (2020)<br><br>China	A-PRF, I-PRF and CGF was isolate and	They were evaluated for the release of PDGF-AB, VEGF, BMP-2	C, CGF; and	Thirty-two blood samples were collected from 8 healthy donors

	fixed for analyse; and<br><br>Evaluation of guided tissue regeneration in periodontal intraosseous defects	and TGF-β1 at the indicated times; and<br><br>15 patients undergone guided tissue regeneration (GTR) with or without A-PRF/CGF were retrieved	T, A-PRF	A-PRF: 200 G for 8 minutes<br><br>CGF: 645 G for 4 minutes
Pitzurra et al, (2020)<br><br>Netherland	Periodontal fibroblast	L-PRF and A-PRF supernatants were added to the experimental plates, and the fibroblasts were incubated. Cell migration, cell proliferation and cell viability were measured	T, A-PRF, and<br><br>C, L-PRF	Membrane were prepared from eight patients and incubated<br><br>A-PRF: 208 G for 8 minutes<br><br>L-PRF: 708 G for 12 min

Esfahood et al, (2020)<br><br>Iran	Cultures were washed with sterile phosphate buffered saline (PBS) twice, and cell proliferation and viability was assessed using the methyl thiazolyl tetrazolium (MTT) assay.	Evaluation of viability and migration of human gingival fibroblasts	C, L-PRF,<br><br>T, A-PRF	Blood collected from two healthy volunteers<br><br>A-PRF: 100 G por 14 minutes<br><br>L-PRF: 700 G for 12 minutes

LEGEND: CGF (Concentrated growth factor); A-PRF (Advanced platlet-rich fibrin); L-PRF (Leucocyte platlet-rich fibrin); PRF (Platlet-rich fibrin); PRP (Platlet-rich plasma); PRGF (plasma rich in growth factors); RPM (rotation per minute).
